# Moderators of pre-post changes in school-based mental health promotion: Psychological stress symptom decrease for adolescents with mental health problems, knowledge increase for all

**DOI:** 10.3389/fpsyt.2022.899185

**Published:** 2022-08-04

**Authors:** Laya Lehner, Vera Gillé, Sabrina Baldofski, Stephanie Bauer, Katja Becker, Silke Diestelkamp, Michael Kaess, Jennifer Krämer, Sophia Lustig, Markus Moessner, Christine Rummel-Kluge, Rainer Thomasius, Heike Eschenbeck, the ProHEAD Consortium

**Affiliations:** ^1^Department of Educational Psychology and Health Psychology, University of Education Schwäbisch Gmünd, Schwäbisch Gmünd, Germany; ^2^Department of Psychiatry and Psychotherapy, Medical Faculty, Leipzig University, Leipzig, Germany; ^3^Center for Psychotherapy Research, University Hospital Heidelberg, Heidelberg, Germany; ^4^Department of Child and Adolescent Psychiatry, Psychosomatics and Psychotherapy, University Hospital of Marburg and Philipps-University Marburg, Marburg, Germany; ^5^Center for Mind, Brain and Behavior (CMBB), Philipps-University Marburg and Justus Liebig University Giessen, Marburg, Germany; ^6^University Hospital Hamburg-Eppendorf, German Center for Addiction Research in Childhood and Adolescence, Hamburg, Germany; ^7^Department of Child and Adolescent Psychiatry, Centre for Psychosocial Medicine, University Hospital Heidelberg, Heidelberg, Germany; ^8^University Hospital of Child and Adolescent Psychiatry and Psychotherapy, University of Bern, Bern, Switzerland; ^9^Institute of Psychology, University of Heidelberg, Heidelberg, Germany; ^10^Department of Psychiatry and Psychotherapy, University Leipzig Medical Center, Leipzig, Germany

**Keywords:** universal prevention, mental health promotion, stress symptoms, mental health literacy, school, adolescence, gender, ProHEAD

## Abstract

**Background:**

School-based mental health promotion aims to strengthen mental health and reduce stress. Results on the effectiveness of such programs are heterogeneous. This study realized a school-based mental health promotion program (*StresSOS*) for all students and aimed to identify moderators (mental health status, gender, grade level) of pre- to post-changes in stress symptoms and knowledge.

**Methods:**

Participants were *N* = 510 adolescents (from 29 classes; 46.7% female) aged 12–18 years (*M* = 13.88, *SD* = 1.00; grade levels 7–10). They were without mental health problems (65.9%), at risk for mental health problems (21.6%), or with mental health problems (12.5%) and participated in a 90 min per week face-to-face training with 8 sessions in class at school. Demographic variables, mental health status, stress symptoms, and knowledge about stress and mental health were collected at baseline. Program acceptance, stress symptoms, and knowledge were collected post-intervention. Multilevel mixed effects models were conducted with the fixed effects time (within factor), mental health status, gender, and grade level (between factors). Random effects for students within classes were included.

**Results:**

In the pre-post comparison, mental health status moderated the changes on psychological stress symptoms (*p* < 0.05). In adolescents with mental health problems the largest reduction in stress symptoms was observed between pre- and post-assessment. Gender and grade level were less relevant. For all adolescents knowledge gains were revealed (*p* < 0.001). Program acceptance was moderated by mental health status and grade level (*p* < 0.01). Mentally healthy adolescents and within the group of adolescents at-risk or with mental health problems, especially younger students (7^th^/8^th^ grade), rated program acceptance higher.

**Conclusion:**

Psychological stress symptoms decreased among adolescents with mental health problems and not among adolescents at risk for or without mental health problems. Mental health-related knowledge increased for all adolescents. The results add to knowledge on school-based mental health intervention research and practice. Its implications for different prevention strategies (universal, selective or a combination of both) are discussed.

## Introduction

Consistent for decades, about one-sixth to one-fifth of all children and adolescents suffer from mental health problems (1, 2). The consequences of mental health problems are often dramatic: not only the adolescents themselves, but also siblings, parents or other reference persons are often severely impaired [e.g., (3)]. In addition, adolescents who suffer from mental health problems have difficulties taking important developmental tasks that would be necessary for healthy, satisfied and successful growing up (4). The problems affect emotions, behavior and thoughts. Thus, in addition to the psychological distress, a healthy development is impeded.

To prevent or reverse the direction of the downward spiral, research efforts have been underway to promote adolescent mental health and teach knowledge about stress/mental health and coping skills [e.g., (5–9)]. The WHO defines mental health as a “state of wellbeing in which the individual is able to realize his or her potential, cope with the normal stresses of life, work productively and fruitfully, and is able to contribute to his or her community” [(10), p. 12]. Achieving mental health can be supported with (school-based) mental health promotion and prevention programs. Adolescence as a phase in which most mental illnesses begin is particularly suitable for the implementation of such programs (11). The school as a place where all children and adolescents can be reached has been repeatedly described as an ideal setting (7, 12, 13).

A prominent conceptual approach is the demands-resources model [e.g., (14, 15)] that defines stress as demands that individuals appraise as significant for wellbeing and as taxing or exceeding resources. Relating the demands-resources perspective to mental health (15), the mental health status of each individual is the result of adaptation and regulation processes between an individual and his or her environment. If coping with demands succeeds, positive emotions and life satisfaction follow; if it does not succeed, negative emotions, stress, dissatisfaction, and in the longer term maladjustment (e.g., mental health problems) can be the result (15). Consequently, mental health promotion programs focus on strengthening resources and skills to enable individuals to deal with the demands [e.g., (16), for the life skills approach]. In this line, *StresSOS* (17), a school-based mental health promotion program targeting adolescents, focuses on enhancing mental health resources and skills by teaching stress management, problem solving, emotion regulation, and mental health literacy to achieve a more favorable balance between demands and resources.

In the past, on the one hand several meta-analyses showed that mental health promotion programs have the potential to increase knowledge, strengthen resources, and reduce stress symptoms [e.g., (13, 18, 19)]. On the other hand, there are also meta-analytical results showing that they are not effective in some cases [e.g., (7)]. What the meta-analyses agree on, however, is that there is high heterogeneity between primary studies. These partly contradictory results lead to the question of whether there are certain moderators that affect the effectiveness of the programs. Identifying moderators could help to generate knowledge about choosing promising target populations and the appropriate prevention strategy in the school setting. Thus, it can be concluded when to address all adolescents (universal prevention) and when it is advisable to implement a program only with certain adolescents (selective prevention). In this way, costs and school capacity could be saved. Following on from these considerations, this paper investigated the participant characteristics mental health status, gender, and grade level as possible moderators of pre-post changes in stress symptoms and knowledge.

According to the demands-resources model, it is conceivable that adolescents with a greater lack of resources (in relation to demands and stressful events) benefit more from interventions promoting these resources. Evidence that this may be the case comes from studies comparing universal and selective programs in their effectiveness [e.g., (13, 19)]. It can be assumed that in the case of selective programs, adolescents have a less favorable constellation of demands and resources, as stress experience plays an important role in the development and the maintenance of mental disorders [e.g., (20, 21)]. In addition, there is evidence that adolescents with mental health problems show lower levels of mental health literacy (22). Within the demands-resources model, this deficit in knowledge and understanding of mental health may represent decreased resources, too. Benefits following mental health promotion and resources strengthening for populations at risk are symptom decrease and the prevention of a worsening of psychological symptoms; for healthy individuals mainly maintaining wellbeing and preventing mental disorders. Thus, in a recent meta-analysis (13), school-based interventions were found to be effective in reducing psychological stress symptoms only in risk groups and not in universal samples. This is in line with the findings of a previous meta-analysis by Beelmann et al. (19) documenting weaker effects for universal programs. Thereby, the considered outcome variables were broad and included among others improvements in symptoms and increases in knowledge.

Following this reasoning, more recent studies conducted for example additional analyses only with students with elevated baseline psychopathology scores since for the total unselected sample within a universal school-based mental health promotion program the expected intervention effect was non-significant (23). Also in the context of universal school-based prevention, Ahlen et al. (24) analyzed baseline symptoms as a potential moderator on the efficacy of the prevention program among children. Brincks et al. (25) combined four former trials (i.e., universal prevention groups or selective risk groups) and thus analyzed a pooled sample of adolescents with varying baseline risk levels. Except for Burckhardt et al. (23), the studies confirmed that the interventions were most beneficial for children or adolescents with high symptom levels. In this line the question of whether mental health status moderates pre-post changes will be further elucidated in the present study. Thereby, we will implement a mental health program for all adolescents and operationalize mental health status independent of stress symptom-related pre-post measures.

Another moderator could be gender. It was repeatedly shown that girls report more stress than boys [e.g., (21, 26–28)]. Consequently, following the assumption that higher stress levels are a consequence of a less favorable ratio of demands and resources, it is conceivable that female participants are more likely to benefit from mental health promotion than male participants. On the other hand, in addition to experiencing more stress symptoms, girls also report more mental health knowledge [representing a mental health resource; (29, 30)]. The results of meta-analyses indicating whether gender moderates the effectiveness of mental health promotion are inconsistent. With regard to prevention programs for depression, studies with more female participants resulted in larger effects for symptom decrease (31). Contrary to this finding, however, Beelmann et al. (19) showed a trend toward a more pronounced effectiveness of mental health promotion programs in studies that included more boys. Hence, it is relatively unclear whether gender has an impact on change over time in the context of mental health programs, and if so, whether there are differential effects for symptom-related or knowledge-related outcomes. Furthermore, it seems unclear whether possible gender differences in program effectiveness are related to gender-related differences in mental health status.

With regard to age group (or grade level as proxy), an increase in the experience of stress and psychosomatic symptoms is evident in the course of adolescence. Older students reported stress experiences (e.g., school, leisure and friends, self) and psychosomatic complaints more often than younger students [e.g., (32, 33)]. Concerning knowledge about mental health/illness research shows a more differentiated mental health literacy in older adolescents (34, 35). Thus, on the one hand, it could be more likely that older adolescents benefit more from mental health promotion due to achieving a better demands-resources balance and stronger symptom relief; on the other hand, younger students could benefit due to the development and strengthening of resources [e.g., mental health literacy, coping strategies; (36)] and staying mentally healthy. However, meta-analyses do not suggest that age moderates the effects of mental health promotion among schoolchildren (13, 19). Following these findings and targeting a narrow age range of early and middle adolescence (grade levels 7 to 10), we do not assume that pre-post changes in stress symptoms or knowledge will be moderated by grade level.

To the best of our knowledge, no health promotion intervention study has been undertaken to jointly analyze mental health status, gender and grade level as potential moderators on the effects of school-based mental health promotion. Thus, in the context of the program *StresSOS*, we investigated pre-post changes, and more importantly, whether these factors moderate changes over time. In terms of outcomes, we focused on stress symptoms as well as knowledge about stress and mental health. In addition, program acceptance was recorded. According to the demands-resources model, it was assumed that more favorable changes during the study period in terms of a decline in stress symptoms and knowledge gain will be found in more severely distressed adolescents with mental health problems compared to healthy adolescents. That is, participants with mental health problems will show greater improvement between pre- and post-assessment in comparison with healthy participants. For the gender factor, as shown, no hypothesis could be formulated. Grade level was not expected to moderate changes over time. With regard to program acceptance, no differences in acceptability were expected depending on mental health status, gender or grade level. *StresSOS* was developed as a universal mental health promotion program and intended to target adolescents with and without mental health problems, girls and boys, older and younger.

## Methods

### Study design

A pre-post study was conducted in which all adolescents received the mental health promotion program *StresSOS* on site in the classroom. The study was part of the ProHEAD project (Promoting Help-seeking using E-technology for ADolescents), a multi-center consortium investigating e-mental health interventions in children and adolescents [see (37)]. Thereby, *StresSOS* was realized either online or on site face-to-face in school classes (17). Within the online trial, participants received an invitation for one out of five ProHEAD programs (17, 38–41) after completing a computerized screening assessment. Adolescents screened without mental health problems were invited to participate in the ProHEAD prevention program *StresSOS* online (not further described here). Within the face-to-face trial, all students of a school class (regardless of their mental health status) participated in the program *StresSOS*. The present study refers to pre-post data of *StresSOS* face-to-face and could thus consider adolescents without mental health problems, those at risk for mental health problems, and those with mental health problems (see below: Participants and Measures).

The prevention program *StresSOS* with 8 weekly 90-min sessions was conducted in German secondary schools. Schools within a 30 km radius of Schwäbisch Gmünd were invited to participate and school classes took part if the teachers wanted to. For the study, a mandatory intervention was conducted for the whole class and a voluntary (i.e., the own and the consent of the parents presupposed) pre-post survey. There were no other inclusion or exclusion criteria: If the principal and class teacher agreed to the participation of a class in *StresSOS*, all students in grades 7 through 10 (minimum age: 12 years) were invited to participate. All students who provided informed consent (own and parental) were eligible to participate.

The survey took place at school, online on a PC. Socio-demographic variables, mental health status, and stress measures (knowledge, symptoms) were collected before the intervention, program acceptance and stress measures were again collected in the last session (post survey). The post survey took place between 8 and 15.6 weeks after baseline (*M* = 11.32 weeks, *SD* = 2.89). The time span for the implementation of the eight sessions varied slightly, because during some *StresSOS* programs there were school vacation weeks in between. The baseline of the ongoing study took place between January 2019 and October 2020. The study was approved by the Ethics Committee of the University of Education Schwäbisch Gmünd.

### Program *StresSOS*

The *StresSOS* program provides content on stress, coping, and mental health literacy; modules are (Session 1) The basics of stress and wellbeing, (Session 2) Managing stress: problem solving, (Session 3) Helpful thoughts, (Session 4) Time to chill out and relax, (Session 5) Upward spirals of positive emotions, (Session 6) Seek support and talk, (Session 7) Mental health and mental illness, (Session 8) A glimpse into the future – finding one's own goals [see (17), for a more detailed description]. The intervention was mainly conducted by trained research assistants and always presented in pairs. Interactive exercises, quizzes, small group work, short inputs or creative methods were used to convey the contents. A voluntary task for home use was also offered.

### Participants

In total, 685 students out of 29 classes from six schools participated in the prevention program, with class sizes varying from 13 to 30. Of these, *N* = 576 (84.1%) agreed to participate in the survey. Sixty-six cases were not included in the analyses for different reasons (*n* = 52 absence from post data collection due to illness, *n* = 11 premature termination of answering the questionnaire, *n* = 3 implausible values). This resulted in a final sample size of *N* = 510 (74.5% of the total sample; 88.5% of the baseline sample). In 499 cases (97.8%), the values were complete. The remaining 11 cases contained missing values which occurred mainly toward the end of the questionnaire due to incomplete questionnaire processing. These missing values were consistently more than 28% per questionnaire, which is why no replacement procedure was used and why there may be deviations from the total sample sizes in individual calculations.

The sample description is shown in Table 1. The students (53.3% boys) were from grade 7 (17.3% of the sample; age: *M* = 12.81 years, *SD* = 0.68), grade 8 (50.6%; age: *M* = 13.64, *SD* = 0.69), grade 9 (19.6%; age: *M* = 14.73, *SD* = 0.66), and grade 10 (12.5%; age: *M* = 15.00, *SD* = 0.74). Overall, their age ranged between 12 and 18 years, with a mean age of 13.88 years (*SD* = 1.0). Participants attended German secondary schools: *Werkrealschule* (19.2%), *Realschule* (30.4%), *Gemeinschaftsschule* (10.2%; secondary schools leading to the lower or intermediate school leaving certificate), and *Gymnasium* (40.2%, secondary school leading to the higher education/university entrance qualification). Three percent of the students came from low socioeconomic status families, 19% from middle affluent families, and 78% from high affluent families. To obtain sufficiently large subgroups, grades 7 and 8 as well as grades 9 and 10 were combined; for socioeconomic status (SES) low or middle affluence vs. high affluence (see Table 1). Based on meeting defined cut-off scores for mental health problems in the baseline screening [see (38)], students were divided into three groups: without mental health problems (65.9%), at risk for mental health problems (21.6%), and with mental health problems (12.5%).

**Table 1:** Sample description.

**Factors**	***n* (%)**
Gender	
Female	238 (46.7)
Male	272 (53.3)
Grade level	
Grades 7 and 8	346 (67.8)
Grades 9 and 10	164 (32.2)
Mental health status	
With mental health problems	64 (12.5)
At risk for mental health problems	110 (21.6)
Without mental health problems	336 (65.9)
School type	
WRS, RS, GS	305 (59.8)
Gymnasium	205 (40.2)
Socioeconomic status	
Low or medium affluence	111 (21.8)
High affluence	399 (78.2)

*N = 510. School type: WRS, Werkrealschule; RS, Realschule; GS, Gemeinschaftsschule (secondary schools leading to the lower or intermediate school leaving certificate); Gymnasium (secondary schools leading to the higher education/university entrance qualification)*.

#### Drop-out analysis

Based on available data, cases that were excluded from the analyses (*n* = 66) were compared with cases that were included (*n* = 510). There were no significant group differences between the excluded and the included data for mental health status (*p* = 0.14), grade level (*p* = 0.42), gender (*p* = 0.15), and school type (*p* = 0.28). Adolescents from low or medium affluent families were overrepresented in excluded cases (*p* < 0.001).

### Measures

#### Socio demographics

Gender, age, grade level, school type, and SES were recorded in the baseline screening. The Family Affluence Scale [FAS; (42), for a more detailed description, see (17)] was used as SES variable (low FAS scores 0–2, medium FAS scores 3–5, high FAS scores > 5).

#### Mental health status

The baseline screening for mental health included the following assessments [for a more detailed description, see (17)]: Emotional and behavioral problems were assessed with the Strengths and Difficulties Questionnaire [SDQ; (43)] with the subscales emotional symptoms, conduct problems, hyperactivity/inattention, and peer relationship problems (20 items). Disordered eating behaviors were assessed by Body Mass Index (BMI), the Short Evaluation of Eating Disorders [SEED; (44)] which assesses key eating disorders symptoms (6 items), and the Weight Concerns Scale [WCS; (45)] that identifies concerns about body weight and physical appearance (5 items). The Patient Health Questionnaire-9 modified for Adolescents [PHQ-A; (46)] was used to assess suicidality and depressive symptoms (9 items). The Car, Relax, Alone, Forget, Friends, Trouble questionnaire [CRAFFT-d; (47)] and the Alcohol Use Disorders Identification Test [AUDIT; (48)] were used to assess risky alcohol consumption. All questionnaires used were available in German.

Using defined cut-off scores [see (38)]^1^, participants were assigned to one of three mental health status groups: without mental health problems, at risk for mental health problems, or with mental health problems. The cut-off scores were WCS <58 and CRAFFT-d <2 and PHQ-A <10 for those without mental problems, WCS > 57 or CRAFFT-d ≥ 2 or PHQ-A > 9 for those at risk for mental problems, and BMI <5 (age/gender percentile) and fear of weight gain (SEED Item 3 > 3), suicidality (PHQ Item 9 = 4), AUDIT ≥ 20, PHQ-A > 14, SDQ total ≥ 20 for those with mental health problems.

#### Stress symptoms

Stress-related symptoms were assessed using the scales somatic symptoms (6 items) and psychological symptoms (12 items; covering three subscales with four items each: anger, sadness, and anxiety) from the Stress and Coping Questionnaire for Children and Adolescents [SSKJ 3-8 R; (49)]. Participants reported the frequency of stress symptoms experienced in the last week on a three-point scale from 1 = *not at all* to 3 = *several times*. The internal consistencies (Cronbach's alpha/McDonald's omega) were 0.89/0.89 (t1) and 0.89/0.89 (t2) for psychological symptoms (total score), 0.83/0.83 (t1) and 0.83/0.83 (t2) for the subscale anger, 0.83/0.84 (t1) and 0.81/0.81 (t2) for sadness, 0.70/0.69 (t1) and 0.76/0.77 (t2) for anxiety, and 0.78/0.78 (t1) and 0.78/0.78 (t2) for somatic symptoms.

#### Knowledge about stress and mental health

A multiple-choice questionnaire with 15 questions was used to assess knowledge about stress, coping strategies, mental health/illness and help seeking. For each item, there were four response options that should be answered as true or false (resulting in a value between zero and four correct responses per item). The knowledge score was assessed as the number of correct answers (resulting in a total score between 0 and 60 for all 15 items). The internal consistencies (Cronbach's alpha/McDonald's omega) were 0.75/0.74 (t1) and 0.83/0.83 (t2).

#### Program acceptance

Acceptance of the program was measured by three items that asked whether the students learnt anything, whether they would recommend the program to a friend, and what grade they would give it. The first two items were answered with four-point scales ranging from 1 = *no, definitely not* to 4 = *yes, definitely*, the last was answered with school grades (1 = *very good* to 5 = *poor*), which were inverted for the analysis. All items were transformed to a range of values from 0 to 10, summed, and then divided by the number of items. In this way, a weighted sum score could be formed, which can assume values between 0 and 10. The internal consistency (Cronbach's alpha/McDonald's omega) was 0.87/0.87.

### Statistical analysis

To account for cluster sampling, we inspected the intraclass correlation coefficient (ICC) and the design effect that is 1 + ICC (average number of students per class – 1). The ICC was calculated at the class level (number of groups = 29, average size = 17.6 students) as sample size was too small at the school level. Design effects ≤ 2 are considered small (50, 51). The ICCs and design effects were rather low for stress symptom measures and higher for knowledge and acceptance: psychological symptoms (total score) ICC/design effect = 0.06/1.997 (t1) and 0.079/2.314 (t2); anger 0.06/1.986 (t1) and 0.062/2.031 (t2); sadness 0.041/1.682 (t1) and 0.034/1.560 (t2); anxiety 0.039/1.645 (t1) and 0.095/2.576 (t2); somatic symptoms 0.025/1.407 (t1) and 0.001/0.976 (t2); knowledge score 0.362/7.005 (t1) and 0.376/7.243 (t2); program acceptance 0.157/3.542. We therefore generally considered students within classes as random factor in the analyses.

To test our hypotheses, for each of the stress symptom scales (i.e., somatic symptoms, psychological symptoms with the total scale and the three subscales anger, sadness, and anxiety) and for knowledge multilevel analyses were calculated as students were nested within classes. Fixed effects (level 1) were examined using mixed ANOVAs with Satterthwaite's method. The within-subject factor was time (t1/pre-, t2/post-intervention), the between-subject factors were gender, grade level (grades 7 and 8 vs. 9 and 10), and mental health status (with mental health problems vs. at risk for mental health problems vs. without mental health problems). All models included random effects for students within classes (level 2). When statistically significant interaction effects with time were present the simple main effects over time were tested *post-hoc* using separate multilevel analyses for each group. For program acceptance, fixed effects of gender, grade level, and mental health status were tested with a three-way univariate ANOVA with Satterthwaite's method. Random effects for students within classes were included.

To evaluate whether the documented effects persist, we conducted an additional multilevel analysis considering SES (low or middle vs. high affluence) as a fourth between-subject factor given the associations between mental health outcomes and social status [e.g., (52)]. Statistical analyses were performed using R (version 4.0.3).

#### Preliminary analyses

##### Between-subject factors

χ^2^-tests were performed to analyze associations between the moderating factors gender, grade level, mental health status, and SES.

##### COVID-19 pandemic

The majority of the sample participated before the onset of the COVID-19 pandemic (*n* = 391, 76.7%). Six classes from the Gymnasium (*n* = 119) participated during the pandemic (October 2020 to December 2020). To evaluate whether the pandemic modified changes over time (and thus the analyses would have to be adjusted), for stress-related symptoms and knowledge mixed ANOVAs were calculated. As in the main analyses described above, time (pre, post) was included as within-subject factor, mental health status, gender, and in addition COVID-19 (before, during) were included as between-subject factors. Within the school type Gymnasium before COVID-19 only younger students (grade 8) participated. Thus, for these ANOVAs, the subsample that participated during COVID-19 was best possible parallelized; older students (grades 9 and 10) were excluded and only younger students (grade 7) were included (before COVID-19: *n* = 86; during COVID-19: *n* = 55).

## Results

### Preliminary analysis

#### Between-subject factors

Gender did not differ across grade levels (*p* = 0.39) and SES (*p* = 0.80). However, among adolescents at risk for and those with mental health problems, there were more girls than expected; among adolescents without mental health problems, boys predominated, χ^2^(2, *N* = 510) = 14.55, *p* = 0.001. Grade level was not related to SES (*p* = 0.76) and mental health status (*p* = 0.06; tendency for more adolescents with mental health problems or at risk in grades 9 and 10). Adolescents at risk for and with mental health problems were overrepresented in low and medium affluence, χ^2^(2, *N* = 510) = 18.80, *p* < 0.001.

#### COVID-19

The interaction effects between COVID-19 and time were not statistically significant: Psychological symptoms (*p* = 0.568; with the subscales anger, *p* = 0.825; sadness, *p* = 0.291; anxiety, *p* = 0.940), somatic stress symptoms (*p* = 0.540), and knowledge about stress and mental health (*p* = 0.119). Thus, changes over time did not differ between adolescents who participated before the onset of the COVID-19 pandemic and those who participated during the pandemic. Based on these results, we decided that performing the main analyses did not require a control for COVID-19 on t1 to t2 changes.

### Main analyses

#### Stress symptoms

##### Psychological symptoms

**Figure 1:**
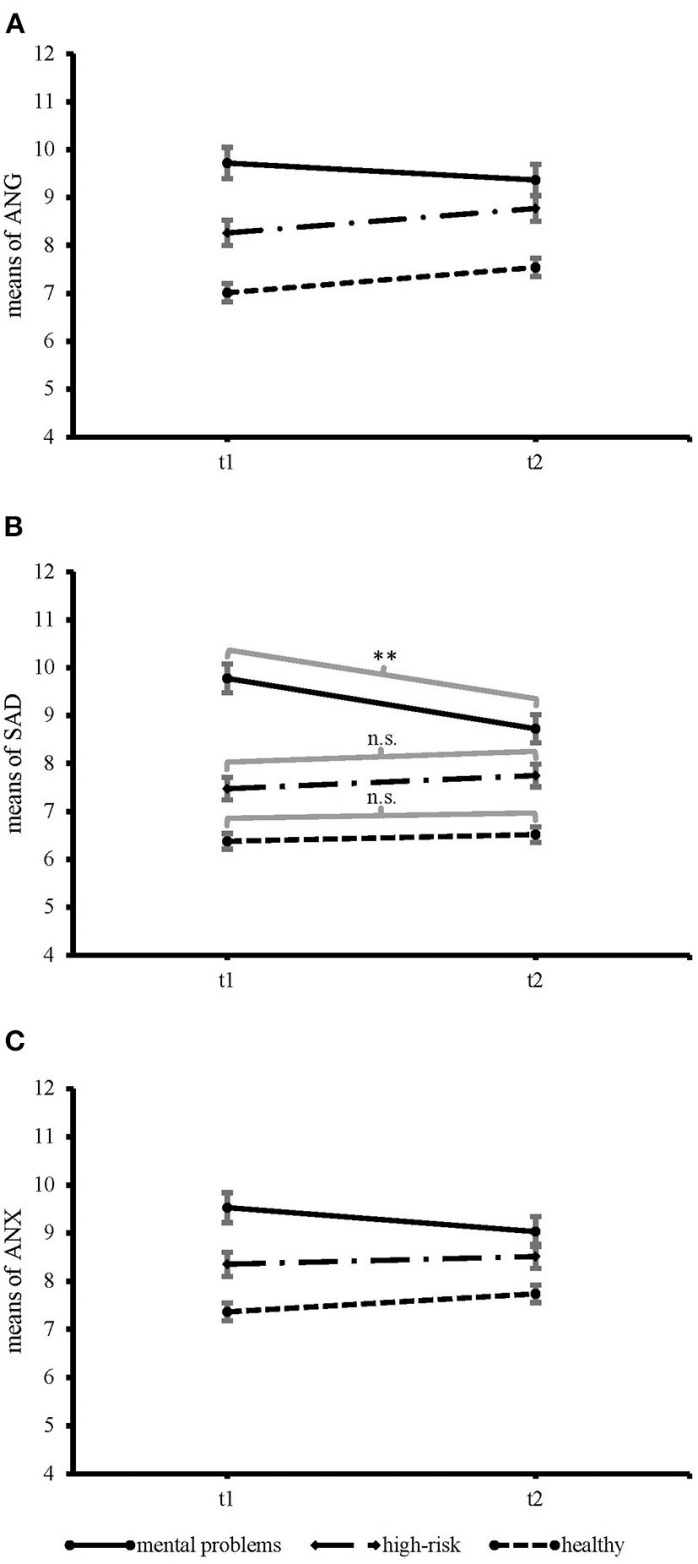
Time × mental health status interaction effect for the psychological symptom subscales. **(A)** anger, *F*(2, 968) = 1.87, *p* = 0.155, **(B)** sadness, *F*(2, 970) = 4.28, *p* = 0.014, and **(C)** anxiety, *F*(2, 968) = 2.01, *p* = 0.135. Error bars mark the standard error of the mean. The possible range of values was between 4 and 12. **p < 0.01.

Results are presented in Table 2. For psychological symptoms, there was no general time effect from t1 to t2 (*p* = 0.949). However, a significant interaction between time and mental health status (*p* = 0.024) revealed that pre-post changes in psychological symptoms were moderated by mental health status: Adolescents with mental health problems (with the highest values overall) showed slightly less symptoms (*M*_t1_ = 29.02, *SE*_t1_ = 0.83, *M*_t2_ = 27.12, *SE*_t2_ = 0.83; *p* = 0.055 two-tailed), no change was seen in adolescents at risk (*M*_t1_ = 23.85, *SE*_t1_ = 0.84, *M*_t2_ = 24.80, *SE*_t2_ = 0.84; *p* = 0.198), and healthy ones (with the lowest values overall) showed symptom increase (*M*_t1_ = 20.78, *SE*_t1_ = 0.45, *M*_t2_ = 21.83, *SE*_t2_ = 0.45; *p* = 0.022). More detailed, with regard to the three subscales, the interaction effect between time and mental health status was particularly evident for sadness (*p* = 0.014) and it was non-significant for the subscales anger (*p* = 0.155) and anxiety (*p* = 0.135). In line with the total scale for psychological symptoms, the pre-post decrease in sadness was evident in adolescents with mental health problems (see Figure 1).

**Table 2:** Summary of fixed effects of the mixed effects model (Satterthwaite's method).

		**Factor**															
		**Within-subject effect and within-between interactions**									**Between-subject effects and interactions**						
**Measure**		**T**	**T × MH**	**T × G**	**T × L**	**T × MH × G**	**T × MH × L**	**T × G × L**	**T × MH × G × L**	**MH**	**G**	**L**	**MH × G**	**MH × L**	**G × L**	**MH × G × L**
PSY	*df*	(1,968)	(2,968)	(1,968)	(1,968)	(2,968)	(2,968)	(1,968)	(2,968)	(2,988)	(1,988)	(1, 43)	(2,989)	(2,988)	(1,988)	(2,990)
*N* = 509	*F*	0.00	3.76	0.07	0.05	0.91	0.39	2.77	0.27	84.30	37.39	1.59	2.17	0.69	0.00	0.51
	*p*	0.949	**0.024**	0.794	0.819	0.402	0.677	0.096	0.761	**<0.001**	**<0.001**	0.214	0.115	0.500	0.957	0.602
ANG	*df*	(1,968)	(2,968)	(1,968)	(1,968)	(2,968)	(2,968)	(1,968)	(2,968)	(2,990)	(1,989)	(1, 43)	(2,991)	(2,990)	(1,990)	(2,991)
*N* = 509	*F*	1.48	1.87	0.08	0.19	0.20	0.09	0.99	0.10	54.81	5.55	3.29	0.28	0.26	0.01	1.60
	*p*	0.225	0.155	0.780	0.661	0.817	0.918	0.321	0.905	**<0.001**	**0.019**	0.077	0.753	0.768	0.929	0.203
SAD	*df*	(1,970)	(2,970)	(1,970)	(1,970)	(2,970)	(2,970)	(1,970)	(2,970)	(2,993)	(1,993)	(1, 46)	(2,994)	(2,993)	(1,993)	(2,994)
*N* = 509	*F*	1.46	4.28	0.28	0.12	0.86	1.58	3.22	0.25	86.62	79.90	0.43	6.23	0.32	0.16	0.76
	*p*	0.227	**0.014**	0.599	0.724	0.424	0.206	0.072	0.779	**<0.001**	**<0.001**	0.516	0.002	0.725	0.687	0.467
ANX	*df*	(1,968)	(2,968)	(1,968)	(1,968)	(2,968)	(2,968)	(1,968)	(2,968)	(2,990)	(1,989)	(1, 43)	(2,991)	(2,989)	(1,989)	(2,991)
*N* = 509	*F*	0.00	2.01	0.03	0.47	1.78	0.45	1.75	0.29	34.75	15.53	0.64	0.54	1.85	0.12	0.45
	*p*	0.945	0.135	0.868	0.492	0.170	0.641	0.187	0.747	**<0.001**	**<0.001**	0.429	0.581	0.158	0.730	0.638
SOM	*df*	(1,969)	(2,969)	(1,969)	(1,969)	(2,969)	(2,969)	(1,969)	(2,969)	(2,988)	(1,995)	(1, 45)	(2,992)	(2,988)	(1,995)	(2,991)
*N* = 510	*F*	4.98	1.61	2.02	0.17	1.52	0.95	0.34	1.47	103.59	53.67	1.08	0.24	1.32	0.73	1.03
	*p*	**0.026**	0.200	0.155	0.681	0.219	0.387	0.559	0.230	**<0.001**	**<0.001**	0.305	0.787	0.269	0.395	0.356
KNO	*df*	(1,968)	(2,968)	(1,968)	(1,968)	(2,968)	(2,968)	(1,968)	(2,968)	(2,974)	(1,976)	(1,66)	(2,975)	(2,974)	(1,976)	(2,974)
*N* = 510	*F*	31.65	0.48	0.16	0.30	0.22	0.91	0.20	0.62	0.83	43.61	1.15	4.10	1.04	7.44	2.00
	*p*	**<0.001**	0.620	0.689	0.586	0.801	0.403	0.653	0.537	0.437	**<0.001**	0.287	**0.017**	0.355	**0.007**	0.135
										**MH**	**G**	**L**	**MH** × **G**	**MH** × **L**	**G** × **L**	**MH** × **G** × **L**
ACC	*df*									(2,473)	(1,471)	(1, 42)	(2,473)	(2,473)	(1,472)	(2,474)
*N* = 499	*F*									1.92	0.56	3.55	2.04	5.12	3.75	1.49
	*p*									0.148	0.456	0.067	0.131	**0.006**	0.053	0.228

*PSY, psychological symptoms; ANG, anger; SAD, sadness; ANX, anxiety; SOM, somatic symptoms; KNO, knowledge about stress and mental health; ACC, program acceptance; T, time (pre, post); MH, mental health status (with mental health problems, at risk for mental health problems, without mental health problems); G, gender (female, male); L, grade level (7^th^/8^th^ grade, 9^th^/10^th^ grade). Random effects for students within classes were included (level 2). p-values <0.05 are bold*.

When SES was added, the pattern of results remained similar: the interaction of time and mental health status was confirmed for the total scale, as well as the subscale sadness (*p*s <0.05; Supplementary Table 1). No statistically significant interaction effects with time and SES resulted (*p*s > 0.35).

Furthermore, there were main effects for mental health status and gender (*p*s <0.001). As expected, adolescents with mental health problems (*M* = 28.09, *SE* = 0.61) experienced more psychological symptoms than those at risk (*M* = 24.55, *SE* = 0.52); in turn, those at risk reported more symptoms than those without mental health problems (*M* = 21.27, *SE* = 0.42). This pattern resulted for the three subscales anger, sadness, and anxiety (*p*s <0.001; see Table 2; Figure 1). Girls reported more symptoms (*M* = 26.02, *SE* = 0.45) than boys (*M* = 23.26, *SE* = 0.48). The gender difference was particularly evident for sadness and anxiety (*p*s ≤ 0.001; Table 2). Further, there was an interaction of mental health status and gender for sadness, showing that especially among adolescents with mental health problems the gender difference was most pronounced (with mental health problems: *M*_*girls*_ = 10.48, *SE*_*girls*_ = 0.27; *M*_*boys*_ = 8.02, *SE*_*boys*_ = 0.27; at risk for mental health problems: *M*_*girls*_ = 8.32, *SE*_*girls*_ = 0.22; *M*_*boys*_ = 6.91, *SE*_*boys*_ = 0.25; without mental health problems: *M*_*girls*_ = 6.91, *SE*_*girls*_ = 0.18; *M*_*boys*_ = 5.99, *SE*_*boys*_ = 0.15).

#### Somatic symptoms

For somatic symptoms, there was a slight general increase from t1 to t2 (*M*_t1_ = 11.29, *SE*_t1_ = 0.18, *M*_t2_ = 11.81, *SE*_t2_ = 0.18; *p* = 0.026). Interaction effects with mental health status, gender or grade level did not reach significance (all *p*s > 0.15; Table 2). Including SES, the main effect for time was no longer statistically significant (*p* = 0.13; Supplementary Table 1), interaction effects with time were also not found.

Further, main effects for mental health status and gender emerged (*p*s <0.001; Table 2). More somatic symptoms were reported from adolescents with mental health problems (*M* = 13.52, *SE* = 0.28) than those at risk (*M* = 11.52, *SE* = 0.22), and the latter showed more symptoms than healthy students (*M* = 9.61, *SE* = 0.15). Girls experienced more somatic symptoms (*M* = 12.41, *SE* = 0.17) than boys (*M* = 10.68, *SE* = 0.19). Adding SES, 7^th^/8^th^ grade students from families with low/medium SES reported more somatic stress symptoms than 9^th^/10^th^ grade students (*M*_7/8_ = 12.12*, SE*_7/8_ = 0.27; *M*_9/10_ = 10.90*, SE*_9/10_ = 0.38). Students from high SES families showed no difference in physical symptoms between grades (*M*_7/8_ = 11.40*, SE*_7/8_ = 0.22; *M*_9/10_ = 11.58*, SE*_9/10_ = 0.25).

#### Knowledge about stress and mental health

There was a significant increase in knowledge from t1 to t2 (*M*_t1_ = 40.19, *SE*_t1_ = 1.02, *M*_t2_ = 42.99, *SE*_t2_ = 1.02; *p* < 0.001). This time effect was not moderated by mental health status, gender or grade level (all *p*s > 0.58; Table 2). When additionally including SES, the main effect for time was unchanged (*p*s <0.001; Supplementary Table 1) and it also was not moderated by SES (*p* > 0.84).

Further, girls (*M* = 43.28, *SE* = 1.02) achieved higher knowledge scores than boys (*M* = 39.90, *SE* = 1.03; *p* < 0.001). An interaction between mental health status and gender (*p* = 0.017) revealed that the gender difference in knowledge was less in mentally healthy adolescents (*M*_*girls*_ = 42.79, *SE*_*girls*_ = 1.05; *M*_*boys*_ = 41.12, *SE*_*boys*_ = 1.01) than it was the case in those at risk (*M*_*girls*_ = 43.16, *SE*_*girls*_ = 1.12; *M*_*boys*_ = 39.57, *SE*_*boys*_ = 1.16) or with mental health problems (*M*_*girls*_ = 43.88, *SE*_*girls*_ = 1.20; *M*_*boys*_ = 38.99, *SE*_*boys*_ = 1.30). Further, the gender difference was less in 7^th^/8^th^ grade students (*M*_*girls*_ = 41.69, *SE*_*girls*_ = 1.11; *M*_*boys*_ = 39.71, *SE*_*boys*_ = 1.17) than it was in 9^th^/10^th^ grade students (*M*_*girls*_ = 44.87, *SE*_*girls*_ = 1.54; *M*_*boys*_ = 40.09, *SE*_*boys*_ = 1.51). With the addition of SES, the same pattern of results was seen (Supplementary Table 1).

### Program acceptance

Overall program acceptance was moderate (*M* = 3.84, *SE* = 0.13). Main effects for mental health status, gender, or grade level did not show (*p*s > 0.06; Table 2). An interaction between mental health status and grade level (*p* = 0.006) revealed that program acceptance was rated higher by 7^th^/8^th^ grade students with mental health problems or at risk for compared to 9^th^/10^th^ grade students with mental health problems (*M*_7/8_ = 4.70*, SE*_7/8_ = 0.57; *M*_9/10_ = 2.87*, SE*_9/10_ = 0.63) or at risk for (*M*_7/8_ = 4.20*, SE*_7/8_ = 0.40; *M*_9/10_ = 2.84*, SE*_9/10_ = 0.56). In contrast, this difference in program acceptance between younger and older students did not show for adolescents without mental health problems (*M*_7/8_ = 4.02*, SE*_7/8_ = 0.31; *M*_9/10_ = 4.20*, SE*_9/10_ = 0.46). Including SES, the interaction of mental health status and grade level was confirmed at *p* = 0.039. Also in line with the results above, no further significant effects were present (Supplementary Table 1).

## Discussion

This study realized a universal school-based mental health promotion program in early and middle adolescence. The main objective was to investigate whether mental health status (i.e., without mental health problems, at risk or with mental health problems), gender or grade level moderate pre- to post-changes in stress symptoms and knowledge. Although meta-analyses [e.g., (13, 19)] agree that mental health promotion programs have the potential to reduce stress symptoms and to increase mental health-related knowledge, they also call for studying potential factors moderating their effectiveness in more detail. In addition, with reference to demand-resources models [e.g., (15)], it can be assumed that there are groups that benefit more (or show stronger changes in the sense of symptom reduction or knowledge increase) from strengthening resources within a health promotion program because of a greater stress-related imbalance. Before detailed discussion, it must be noted that without a control group changes cannot be causally attributed to the program *StresSOS* and moderators cannot be interpreted as differential intervention effects. However, the current study did not aim at evaluating the effects of *StresSOS* on stress-symptoms and knowledge, but rather providing first insights whether changes between pre- and post-assessment differ between participants of different mental health status, gender or grade level.

### Stress symptoms

In line with the assumptions, in the present study, pre-post change in psychological stress symptoms was moderated by mental health status. Most important, in particular adolescents with mental health problems showed a significant decrease in psychological stress-symptoms, especially for sadness. This decrease was not observed in adolescents at risk or without mental health problems. Based on previous research showing stronger effects in selective interventions for children at risk for mental health problems [e.g., (13)], one could additionally assume that adolescents at risk would show improvements in the sense of a reduction in psychological stress symptoms, too. However, this was not the case in the present study. A potential explanation could be linked to markedly higher cut-offs in psychopathology measures for group assignment and the significantly higher baseline levels for psychological symptoms, both indicating that adolescents with mental health problems were most heavily burdened overall. Compared to normative data for early adolescence from an unselected school-based sample (49), the observed mean values of adolescents with mental health problems indicated a high level of baseline stress symptomatology (around the 84^th^ percentile), for those at risk the stress level on average was moderate (percentiles about 69), and for adolescents without mental health problems stress levels were lowest (around the 40^th^ percentile). Accordingly, our study showed a positive change in the most stressed group. For primary school children, Ahlen et al. (24) also found a decrease in depressive symptoms within a universal prevention program only for children with highest depressive symptoms at baseline (above the 90^th^ percentile of the studied sample), but not for those above the median or the 75^th^ percentile. For the other two groups in our study, adolescents without mental health problems or at risk, the question would be rather longer term, whether their good mental health will maintain and the development of more severe stress symptoms and psychopathology will be prevented; a current reduction of symptoms, however, is less likely to occur due to the low to moderate “asymptomatic” levels.

Regarding somatic stress symptoms, the interaction indicating that more severely distressed adolescents showed a more favorable pre-post change was non-significant. It was a little surprising, because adolescents with mental health problems again reported significantly more symptoms than the two other groups [on average around the 84^th^ percentile compared to normative data; (49)] and thus having the clear potential for symptom decrease. Instead, there was a slight general increase over time for all which however showed less robust when socioeconomic status was added as a control factor. Intervention studies do not regularly distinguish between psychological symptoms and somatic symptoms [e.g., (12), for varied stress outcomes] which would yet be relevant. However, differentiating this, contrary to our findings, Lohaus et al. (53) showed reductions in stress symptoms 1 week and also 2 months after a school-based prevention training only for somatic symptoms and not for psychological symptoms (using the same scales as in the current study). Explanations remain speculative, but it would be interesting to study different time periods and investigate whether changes in somatic and psychological symptomatology tend to be similar, mutually influencing or independent.

It is also worth noting that changes over time in stress symptomatology did not differ regarding gender [despite higher symptom scores for girls; e.g., (21, 26)] and grade level. For gender, the results of recent studies were already inconsistent [e.g., (19, 31)] and for grade level, with early and middle adolescence, we have included a narrow range. Overall, our results highlight the importance of mental health status for pre- to post-changes and less of gender and grade level.

### Knowledge about stress and mental health

For knowledge, in line with previous research [e.g., (53); see also (19), for a meta-analysis] positive pre-post changes were shown. Contrary to our expectation, the increase was not moderated by mental health status. Gender and grade level also did not matter. Although 9^th^/10^th^ grade girls in particular had higher knowledge scores and boys with mental health problems or those at risk showed lower scores. The improvement in knowledge about stress/mental health and coping skills, however, was generally evident for all adolescents. This result is very relevant as it can be assumed that knowledge benefits mental health [e.g., (54), for mental health literacy with knowledge as a central component] and might act as a necessary prerequisite for mentally healthy behaviors. Hence, in the field of public mental health strengthening knowledge and mental health literacy was identified as central topic [e.g., (55)]. On the other hand, knowledge about stress/mental health and coping skills (as frequent in intervention research) was captured very narrow to the content of the program *StresSOS*; this may facilitate detecting improvements beside the fact that knowledge is in itself easier to change than behavior or symptoms. Also knowledge was not constantly confirmed as a powerful predictor for health [e.g., (56), for nutrition-related behaviors]. Therefore, future research could investigate its influence on mental health in adolescents; especially whether increasing knowledge (as shown in the present study) will pave the way for adequate coping in (future) stressful situations and support mental health.

### Program acceptance

With regard to program acceptance, among adolescents at-risk or with mental health problems younger students (7^th^/8^th^ grade) rated the program acceptance higher than older students (9^th^/10^th^ grade). This difference did not show for adolescents without mental health problems. Reasons for the lower program acceptance of older mentally burdened adolescents should be investigated more closely in the future. Possibly, the program content fitted better to the younger; so that an early intervention and additionally a grade level-appropriate and health sensitive program modification could be advisable. Moreover, there was no gender difference indicating that *StresSOS* equally addressed boys and girls in line with the general demand for sensitive mental health strategies [e.g., (16)].

### Limitations and strengths

Our study has some limitations that must be noted. First, without a control group the effectiveness of the *StresSOS* training cannot be examined and the observed time effects cannot be reliably attributed to the intervention. Thus, spontaneous remission or regression to the mean could be possible explanations for the observed decrease in psychological stress symptoms among adolescents with mental health problems. However, from a clinical perspective, the prevalence of mental disorders is high in adolescence [with an increase into young adulthood; e.g., (57)]. Hence, among the mentally burdened group, a worsening would rather be the expected course than an improvement. For future studies, however, an enhanced design with a control group or repeated pretests would be valuable. Second, the studied groups differed in sample size which can have an impact in terms of power. In particular, this could be relevant for the group of adolescents with mental health problems. On the one hand, this was the smallest group (*n* = 64), however, stronger effect sizes can be assumed for this group in particular. Related hereto, the studied factors were not completely independent of each other. Thus, in line with literature lower socioeconomic status was associated with poorer mental health [e.g., (58)]. Also adolescents from families with lower SES were overrepresented among the cases excluded for analyses (mainly due to illness on the day of the survey). While associations between SES and school absenteeism were documented (59), evidence for a direct link to mental health was limited. With regard to gender, internalizing problems are more likely in girls, externalizing behavior problems in boys (2). In the current study, girls reported more mental health problems even though the mental health status was captured comprehensively including, for example, emotional problems or eating pathology as well as substance use or conduct problems. Overall, a larger sample size could be advantageous to evaluate the partially emerging interactions with higher confidence. Third, data were assessed pre- and post-intervention covering a short time interval of about 11 weeks. Follow up data to analyze longer-term changes were not considered and would be an important point of future research. Fourth, most students participated in the intervention before the COVID-19 pandemic, about one fifth (exclusively from the school type Gymnasium) at the time when the “second wave” started. Even if there was no evidence of bias in the preliminary analyses, an influence cannot completely be ruled out. Finally, the present study focused on individual characteristics as moderators of pre-post changes, but contextual factors such as program implementation or even both factors in interaction also affect outcomes of health promotion programs [e.g., (60)]. Future school-based intervention studies may benefit from including the role of the school setting for mental health promotion (e.g., facilitators or barriers for implementation, health promotion policy).

Major strengths of this study are the pre-post design to study changes over time and the focus on potential moderating factors. In particular, the comparison between different mental health statuses accompanied by the comprehensive baseline data collection for group assignment is a unique feature of the study. Also the sample size is appropriate for the present analyses and considerably larger than most other studies on mental health promotion in schools [for example (13)]. The overall participation rate of 84% was satisfying and for 89% of the baseline sample post-data were available.

## Conclusion

In conclusion, our study offers insights in individual factors moderating pre- to post-changes in stress symptoms and knowledge about stress and mental health that will be valuable in the context of future intervention research. While mental health status was related to stress symptom decrease, associations with both gender and grade level and with the additional control factor socioeconomic status were less relevant. For knowledge, all students showed an improvement. Mentally healthy adolescents and within the group of adolescents at-risk or with mental health problems, especially younger students (7^th^/8^th^ grade), rated program acceptance higher.

All in all, our finding may help to decide in school-based health promotion whether a universal or a selective strategy should be implemented. First, a universal strategy always is reasonable when sufficient time and capacities are available. Even if healthy adolescents initially only show knowledge gain, those under greater stress can benefit in terms of symptom reduction, and it can be assumed that longer-term preventive effects will also be seen in adolescents without mental health problems. Second, nevertheless in situations where few capacities are available, a selective strategy, only for adolescents with higher stress, may be recommended [see (13)]. This would also be beneficial in terms of cost-effectiveness (61). Third, a possibly ideal variation, considers combining both strategies universal and selective to achieve optimal outcomes [e.g., (9)]. For example, after screening, adolescents without mental health problems receive a mental health promotion and stress prevention program in the sense of a universal strategy, while adolescents at risk for developing mental illness or with pre-existing problems receive a program tailored to their problems in the sense of a selective strategy. In the ProHEAD project (37) this combination of both strategies universal and selective is implemented and the present study relates to the universal approach. Future studies will then be able to compare the different strategies among the ProHEAD programs and moreover for *StresSOS* the face-to-face program in the classroom (presented here) with an online intervention and examine the benefits and limitations of each.

## Data availability statement

The raw data supporting the conclusions of this manuscript will be made available by the corresponding author to any qualified researcher upon request without undue reservation.

## Ethics statement

The studies involving human participants were reviewed and approved by Ethics Committee of the University of Education Schwäbisch Gmünd. Written informed consent to participate in this study was provided by the participants' legal guardian/next of kin.

## The ProHEAD Consortium

The ProHEAD consortium comprises six study sites in Germany. Site leaders are: Michael Kaess (University Hospital Heidelberg), Stephanie Bauer (University Hospital Heidelberg), Rainer Thomasius (University Medical Center Hamburg-Eppendorf), Christine Rummel-Kluge (University Leipzig), Heike Eschenbeck (University of Education Schwäbisch Gmünd), Hans-Joachim Salize (Medical Faculty Mannheim/Heidelberg University) and Katja Becker (Philipps-University of Marburg). Further members of the consortium are: Katja Bertsch, Sabrina Bonnet, Romuald Brunner, Johannes Feldhege, Christina Gallinat, Stella Hammon, Sabine C. Herpertz, Julian Koenig, Sophia Lustig, Markus Moessner, Fikret Özer, Peter Parzer, Regina Richter, Franz Resch, Johanna Sander (all University Hospital Heidelberg), Steffen Luntz (Coordinating Center for Clinical Trials Heidelberg), Silke Diestelkamp, Anna-Lena Schulz (all University Medical Center Hamburg-Eppendorf), Sabrina Baldofski, Sarah-Lena Klemm, Elisabeth Kohls, Sophia Müller, Lina-Jolien Peter, Mandy Rogalla (all University Leipzig), Vera Gillé, Hanna Hofmann, Laya Lehner (all University of Education Schwäbisch Gmünd), Elke Voss (Medical Faculty Mannheim/Heidelberg University), Alisa Hiery, Jennifer Krämer (all Philipps-University of Marburg).

## Author contributions

LL and HE wrote the first draft of this manuscript. HE is the principal investigator of the StresSOS trial within the ProHEAD Consortium. LL, VG, and HE developed the StresSOS face-to face program. LL and VG managed participant recruitment, intervention delivery, and data collection for the StresSOS face-to-face study. JK, LL, SL, SBal, SD, and VG are the scientific staff for the ProHEAD trial and are critically involved in the realization of the study. SBau and MM are responsible for technological support. KB, SBau, HE, MK, CR-K, and RT obtained funding for the ProHEAD Consortium and took the leading role in designing the study. MK and SBau are the coordinators of the ProHEAD Consortium. All authors contributed to the article and approved the final manuscript.

## Funding

The study is funded by the German Federal Ministry of Education and Research (BMBF) Grant (01GL1744F).

## Conflict of interest

The authors declare that the research was conducted in the absence of any commercial or financial relationships that could be construed as a potential conflict of interest.

## Publisher's note

All claims expressed in this article are solely those of the authors and do not necessarily represent those of their affiliated organizations, or those of the publisher, the editors and the reviewers. Any product that may be evaluated in this article, or claim that may be made by its manufacturer, is not guaranteed or endorsed by the publisher.

## Footnotes

^1^After completion of 10% of the ProHEAD baseline screenings (*n* = 1500) the allocation ratio was evaluated and pre-registered cut-off scores were adjusted.
