# Supplementary material for: Moderators of pre-post changes in school-based mental health promotion: Psychological stress symptom decrease for adolescents with mental health problems, knowledge increase for all
Source: Front Psychiatry. 2022 Aug 4;13:899185. doi: 10.3389/fpsyt.2022.899185 (PMC9387723; doi:10.3389/fpsyt.2022.899185)
Supplement: Supplementary file 1 [file Table_1.pdf]

frontiers

Supplementary Table 1

Summary of fixed effects of the mixed effects model (Satterthwaite's method) controlling for socioeconomic status

		Factor								
		Within-subject effect and within-between interactions					Between-subject effects			
Measure		T	T × MH	T × G	T × L	T × SES	MH	G	L	SES
PSY	*df*	(1,944)	(2,944)	(1,944)	(1,944)	(1,944)	(2,964)	(1,965)	(1,48)	(1,894)
N = 509	*F*	0.07	3.09	0.04	0.30	0.56	79.11	31.55	1.75	0.71
	p	.787	**.046**	.847	.584	.454	**<.001**	**<.001**	.192	.398
ANG	*df*	(1,943)	(2,943)	(1,943)	(1,943)	(1,943)	(2,965)	(1,966)	(1,48)	(1,871)
N = 509	*F*	0.29	1.26	0.02	0.01	0.85	50.69	6.58	3.16	1.06
	p	.588	.284	.883	.906	.357	**<.001**	.010	.082	.304
SAD	*df*	(1,945)	(2,945)	(1,945)	(1,945)	(1,945)	(2,967)	(1,969)	(1,56)	(1,786)
N = 509	*F*	1.54	3.50	0.06	0.86	0.63	80.86	65.38	0.71	0.00
	p	.215	**.030**	.802	.355	.429	**<.001**	**<.001**	.403	.950
ANX	*df*	(1,944)	(2,944)	(1,944)	(1,944)	(1,944)	(2,965)	(1,966)	(1,49)	(1,868)
N = 509	*F*	0.00	2.11	0.33	0.33	0.01	33.24	11.77	0.52	0.38
	p	.979	.122	.568	.564	.914	**<.001**	**<.001**	.476	.538
SOM	*df*	(1,945)	(2,945)	(1,945)	(1,945)	(1,945)	(2,962)	(1,971)	(1,65)	(1,570)
N = 510	*F*	2.26	1.69	1.25	0.05	2.29	84.39	54.59	3.12	0.01
	p	.133	.184	.263	.830	.131	**<.001**	**<.001**	.082	.939
KNO	*df*	(1,943)	(2,943)	(1,943)	(1,943)	(1,943)	(2,952)	(1,959)	(1,58)	(1,972)
N = 510	*F*	26.71	0.15	0.06	0.09	0.04	0.05	30.34	1.15	2.24
	p	**<.001**	.859	.812	.763	.848	.948	**<.001**	.288	.135
							MH	G	L	SES
ACC	*df*						(2,462)	(1,461)	(1,47)	(1,468)
N = 499	*F*						1.09	0.14	3.60	0.60
	p						.339	.708	.064	.440

Note. PSY = *psychological symptoms*; ANG = *anger*; SAD = *sadness*; ANX = *anxiety*; SOM = *somatic symptoms*; KNO = *knowledge about stress and mental health*; ACC = program *acceptance*; T = time (pre, post); MH = mental health status (with mental health problems, at risk for mental health problems, without mental health problems); G = gender (female, male); L = grade level ($7^{th}/8^{th}$ grade, $9^{th}/10^{th}$ grade); SES = socioeconomic status (low/medium, high affluence). Random effects for students within classes were included (level 2). *p*-values < .05 are bold.

Within-subject interaction effects with more than two factors and between-subject interactions were not presented for clarity. Statistically significances with $p < .05$ were present for psychological symptoms: MH × G × L × SES, $F(2, 969) = 3.53$, $p = 030$; for anger: MH × SES, $F(2, 969) = 3.33$, $p = .036$; for sadness: MH × G, $F(2, 968) = 4.57$, $p = .011$; MH × G × L × SES, $F(2, 968) = 6.16$, $p = .002$; for somatic symptoms: L × SES, $F(1, 616) = 6.68$, $p = .010$; for knowledge: MH × G, $F(2, 957) = 3.83$, $p = .022$; G × L, $F(1, 959) = 4.53$, $p = .034$; and for program acceptance: MH × L, $F(2, 461) = 3.26$, $p = .039$.
